# Interleukin 17A Promotes Gastric Cancer Invasiveness via NF-κB Mediated Matrix Metalloproteinases 2 and 9 Expression

**DOI:** 10.1371/journal.pone.0096678

**Published:** 2014-06-06

**Authors:** Yidong Wang, Hong Wu, Xiaoling Wu, Zhuoqiong Bian, Qing Gao

**Affiliations:** 1 Department of Obstetrics and Gynecology, the Second Affiliated Hospital, Medical School of Xi’an Jiaotong University, Xi’an, Shaanxi, P. R. China; 2 Department of General Surgery, XIAN XD GROUP Hospital, Xi’an, Shaanxi, P. R. China; 3 Department 5 of rheumatology, the fifth hospital of Xi’an city, Xi’an, Shaanxi, P. R. China; University of Patras, Greece

## Abstract

Interleukin 17A (IL-17A), as a pro-inflammatory cytokine, is involved in pathology of inflammatory diseases and tumor microenvironment. The aim of this study is to investigate the effect of IL-17A on the invasiveness of gastric cancer (GC). In the study, we found that IL-17A could promote the migration and invasion of GC cells. Furthermore, after treated with IL-17A, the expressions and activities of matrix metalloproteinase 2 (MMP-2) and MMP-9 were upregulated, while the expressions of TIMP-1 and TIMP-2 were downregulated. Moreover, the nuclear/overall fractions of p65 and p50 were dramatically elevated by IL-17A. Pretreatment with helenalin, a nuclear factor-κB (NF-κB) inhibitor, was proved to abolish the promoting effect of IL-17A on the invasion ability of GC cells and upregulation of MMP-2 and MMP-9. In conclusion, our findings illustrated that IL-17A could promote the invasion of GC cells by activating NF-κB pathway, and subsequently upregulating the expression of MMP-2 and MMP-9. These results may lead to the identification of new diagnostic markers and therapeutic targets of GC.

## Introduction

As one of the top causes of cancer-related death worldwide, GC is responsible for over 700,000 deaths per year [1]. It is the second most common cancer and the third leading cause of death among cancer patients in China [2]. The potent invasion and metastasis ability of GC is one of the important factors that lead to poor prognosis [3]. Therefore, it is significant to uncover the underlying mechanisms, to identify novel molecular therapeutic targets that regulate invasion abilities of GC cell, and thus to develop advancing treatments for GC.

IL-17A is the founding member of the IL-17 family which ranges from IL-17A to IL-17F [4]–[8]. IL-17 family members play an active role in various diseases such as autoimmune diseases, inflammatory diseases, and malignant diseases. Persistent inflammation has been considered to be correlated with increased risk of GC [9]. Early steps in gastric carcinogenesis involve *Helicobacter pylori* (H. pylori) infection leading to chronic active and atrophic gastritis with production of proinflammatory mediators [10]–[13], such as IL-1b, TNFα, IFNγ, IL-6, and IL-8. However, the role of IL-17A in cancer was inconsistent according to previous reports. Some evidence revealed that IL-17A might promote tumor growth by stimulating angiogenesis and invasive capacity of tumor cells [14]. In contrast, other studies suggested that IL-17A showed a protective effect against chronic lymphocytic leukemia development by promoting immune system-mediated tumor rejection [15]. Furthermore, IL-17A enhanced the cytotoxic effects of NK cells against tumor cells by augmenting the expression of cytotoxic molecules [16]. Up to now, there is less report about the role of IL-17A in the invasion of GC.

In the present study, we found that IL-17A could increase GC cell motility by upregulating MMP-2 and MMP-9 via activating NF-κB transcript factors.

## Materials and Methods

### Cell Culture

Human GC cell line AGS was purchased from ATCC (American Type Culture Collection, Manassas, VA) and maintained in DMEM supplemented with 10% fetal bovine serum (FBS; Hyclone, Logan, UT), 100 U/L penicillin and 100 mg/L streptomycin. Human GC cell lines BGC-823 and SGC-7901 were obtained from Chinese Academy of Sciences (Shanghai, China), and cultured in RPMI-1640 medium containing 10% bovine serum, penicillin (100 U/mL) and streptomycin (100 µg/mL). All cells were cultured at 37°C, 5% CO_2_ air atmosphere.

### Wound Healing Assay

GC cells were cultured as confluent monolayers in a 6-well plate and were wounded by removing a 1 mm strip across the well with a pipette tip when they adhered to the plate surface. The wounded monolayers were then washed twice with PBS to remove non-adherent cells before 1 ml medium with or without IL-17A (50 ng/mL) (R&D System, Minneapolis, MN) were added. After 12 h incubation, different concentration groups were photographed by inverted micrographs.

### *In vitro* Invasion Assay

The *in vitro* invasion assay was performed using the Bio-Coat Matrigel invasion assay system (Becton Dickinson, Franklin Lakes, NJ) as described previously [17]. After treated with or without IL-17A (50 ng/ml), GC cells were seeded in 200 µl serum-free medium and placed in the upper chambers at 2×10^5^ cells and normal growth medium were placed in underneath chambers. Twenty-four hours later, the cells on the upper surface of the membrane were removed with cotton swabs, while cells on the bottom side of the filter were fixed, stained and measured. The percentage of invasive rate was expressed as a percentage of control.

### Western Blotting Analysis

Whole cell lysates from GC cells were harvested with cell lysis buffer. Nuclear lysates from cultured cells were harvested with NucBusterTM Protein Extraction Kit (Novagen, Germany) according to manufacturer’s instructions. Western blotting analyses were performed with the standard protocol using antibodies against MMP-2, MMP-9, TIMP-1, TIMP-2, p52 and β-actin (Cell Signaling Technology, Beverly, MA), NF-κB-p50, p65/RelA, c-Rel, RelB, and Histone H1 (Santa Cruz Biotechnology, Santa Cruz, CA).

### Zymography

Cells were treated with different concentrations of IL-17A at 37°C for 24 h, and samples of conditioned medium were collected. Appropriate volumes of the samples (adjusted by vital cell number) were separated by 0.1% gelatin-8% SDS-PAGE electrophoresis. After electrophoresis, the gels were washed twice in 2.5% Triton X-100 at room temperature for 30 min and then incubated in reaction buffer (10 mM CaCl_2_, 40 mM Tris-HCl and 0.01% NaN_3,_ pH 8.0) at 37°C for 12 h. Coomassie brilliant blue R-250 gel stain was then used to stain the gel. The intensities of bands on the gels were calculated using an image analysis system (Bio-Rad Laboratories, Richmond, CA).

### Statistical Analysis

All data were expressed as the means ± SD. The statistical analysis was carried out using the SPSS 16.0 software (SPSS Inc., Chicago, Illinois, USA) to evaluate statistical differences. Student’s *t*-test was used for comparisons between two groups and one-way or two-way analysis of variance was used to analyze statistical differences between groups under different conditions. A *p* value less than 0.05 was considered to be statistically significant. All statistical tests were two sided.

## Results

### IL-17A Increases Cell Motility in GC Cells

**Figure 1:**
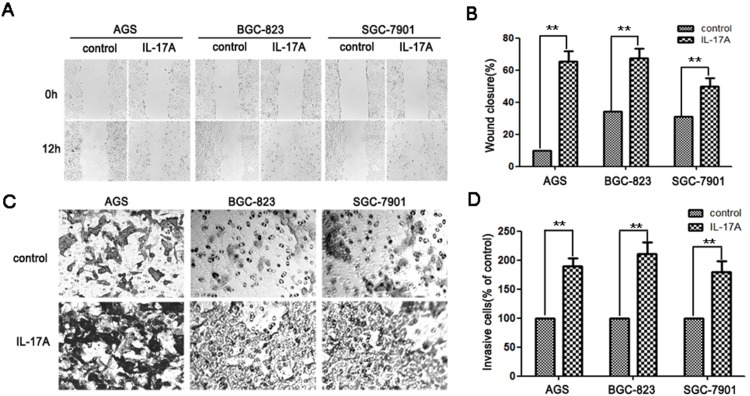
IL-17A promotes gastric cancer cell migration and invasion. (A) IL-17A treated GC cells (AGS, BGC-823 and SGC-7901) showed higher motility in a wound-healing assay, compared with cells without IL-17A treatment. (B) The percent migration rate is expressed as a percentage of the beginning area. (C) Effect of IL-17A on cell invasion was detected by transwell assay. Representative pictures of cells migrated through Matrigel-coated transwell were shown. (D) Total invasive cell number in each chamber was summarized as a percentage of control. Values represent the means ± SD of three independent experiments performed in triplicate. *p<0.05 and **p<0.01 compared with the control group.

A scratch-wound assay was performed to determine the effect of IL-17A on the migration in AGS, BGC-823, and SGC-7901 cells. The extension of the cell migration was quantified by estimating the percentage of recolonization of the wound surface after 24 h. In the control group, the movement was not obvious. In the presence of IL-17A, this movement was significantly promoted after 12 h of incubation (Figure 1A). Quantification analysis indicated that the difference was significant (Figure 1B). Moreover, the invasion assay showed that the invasive ability of IL-17A treated cells was significantly stronger than control parental cells in all three tested cell lines (Figure 1C and 1D). These data showed that IL-17A could enhance GC cell migration and invasiveness. Furthermore, we also observed that IL-17A has no significant effect on the proliferation of BGC-823, SGC-7901 and AGS cells (Figure S1), suggesting that the promoting effect of IL-17A on migration and invasiveness are not interfered by cell proliferation.

### Effect of IL-17A on Upregulation of MMP-2 and MMP-9 and Downregulation of TIMP-1 and TIMP-2 in GC Cells

**Figure 2:**
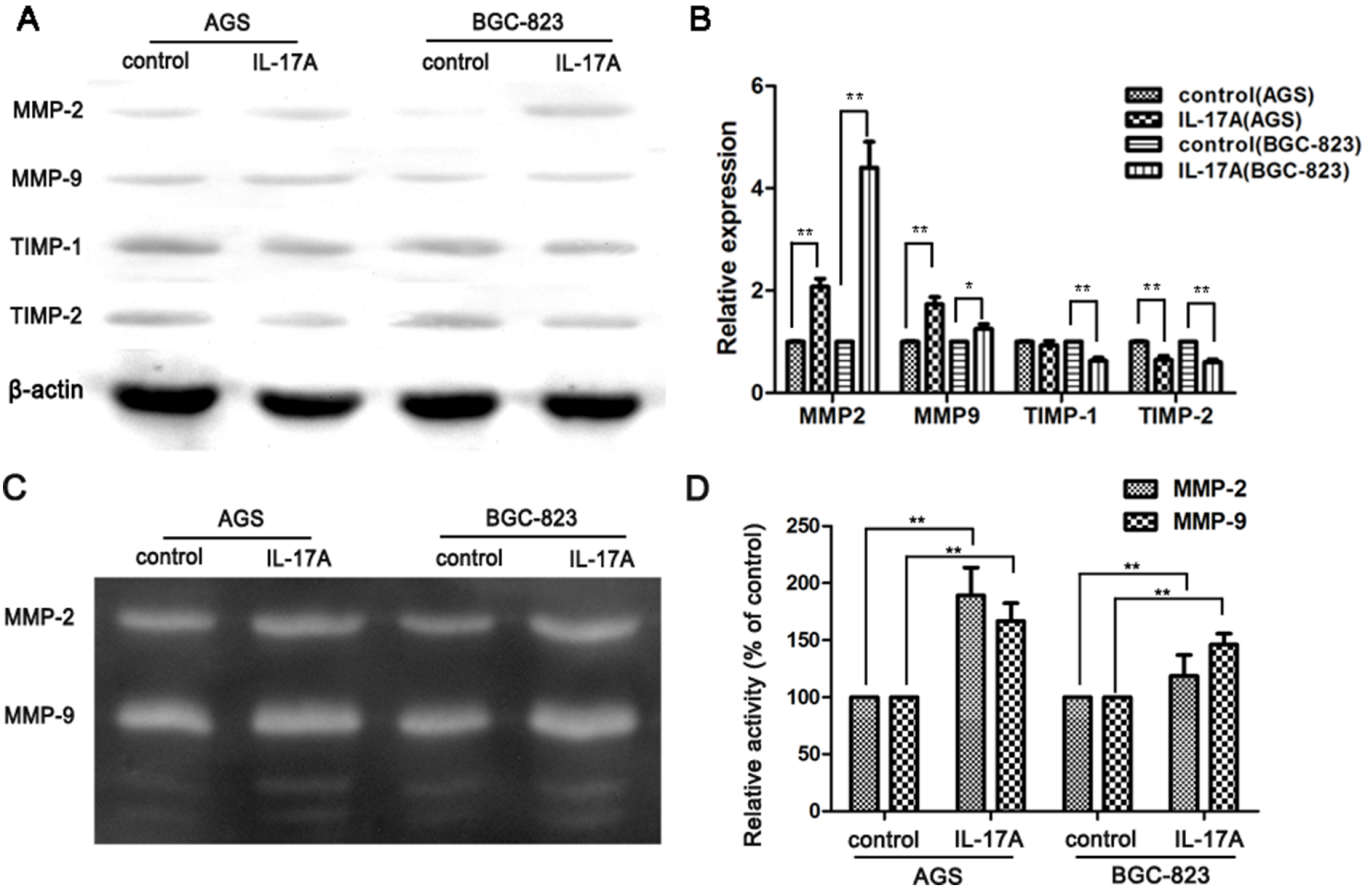
IL-17A promotes the expressions and activities of MMP-2 and MMP-9 and suppresses the expressions of TIMP-1 and TIMP-2 in gastric cancer cells. (A) Expressions of MMPs in GC cells (AGS and BGC-823) were compared by western blotting between cells treated with and without IL-17A (50 ng/ml) for 24 h. (B) Quantification of the protein levels of MMP-2 and MMP-9. (C) Effects of IL-17A on the activities of MMP-2 and MMP-9. (D) Quantification of the activities of MMP-2 and MMP-9. Values represent the means ± SD of three independent experiments performed in triplicate. *p<0.05 and **p<0.01 compared with the control group.

Since overexpression of MMPs can promote cancer metastasis [18], [19], we next investigated the effect of IL-17A on MMPs expression in GC cell lines. As shown in Figure 2 and Figure S2, the expressions of MMP-2 and MMP-9 were compared using Western blotting between IL-17A treated and untreated cells. The result showed that MMP-2 and MMP-9 were upregulated in IL-17A treated cells (AGS and BGC-823) (Figure 2A and 2B). At the same time, the expressions of TIMP-1 and TIMP-2 were downregulated (Figure 2A and 2B). Gelatin zymography was also performed to assess the activity of MMP-2 and MMP-9 in GC cells treated with or without IL-17A. The results showed that IL-17A enhanced the activity of MMP-2 and MMP-9 in GC cells (AGS and BGC-823) (Figure 2C and 2D). Similar results have been observed in SGC-7901 cells (Figure S2). These results suggested that the pro-metastasis effect of IL-17A on GC might be through regulating MMP/TIMP balance and prompted us to further explore its mechanism of action.

### IL-17A Upregulates MMP-2 and MMP-9 Expressions via Activating NF-κB Pathway

**Figure 3:**
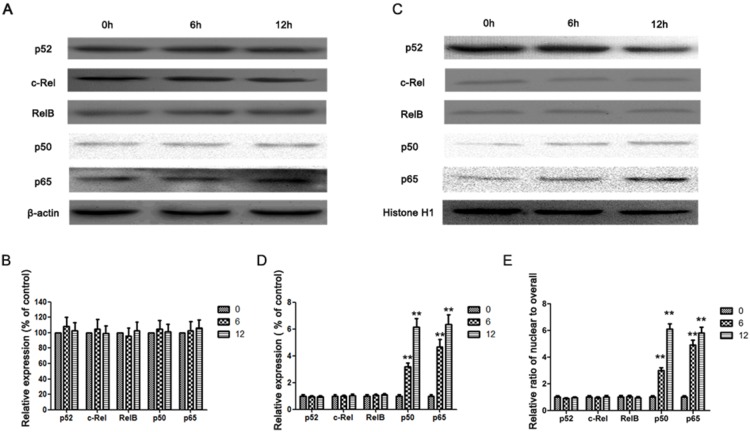
IL-17A activates NF-κB pathway in AGS cells. (A) Western blotting analysis was used to detect overall p50, p65, p52, c-Rel and RelB expression in AGS cells treated with IL-17A (50 ng/ml) at indicated time points. (B) Quantification of the protein levels of overall p50, p65, p52, c-Rel and RelB. (C) Western blotting analysis was used to detect nuclear p50, p65, p52, c-Rel and RelB expression in AGS cells treated with IL-17A (50 ng/mL) at indicated time points. (D) Quantification of the protein levels of nuclear p50, p65, p52, c-Rel and RelB. (E) The relative ratio of nuclear to overall fraction of p50, p65, p52, c-Rel and RelB. Values represent the means ± SD of three independent experiments performed in triplicate. **p<0.01, compared with the control group.

NF-κB has been reported as a downstream target of IL-17A signaling pathway in many cells [17], [20], [21], which is able to upregulate MMP-2 and MMP-9 expressions [14], [17]. And IL-17A was also reported to increase the expression of MMPs via activating NF-κB pathway in many cells [14], [17]. Therefore, we next checked whether IL-17A upregulated MMP-2 and MMP-9 expressions in GC cells through activating NF-κB. As shown in Figure 3, the expression and translocation of NF-κB subunits in nucleus were analyzed by Western blotting. We observed that the level of p65 and p50 in nuclei was dramatically elevated in AGS cells after IL-17A treatment, while the level of overall p65 and p50 has no change (Figure 3A–D). In addition, the nuclear/overall fraction of these molecules was increased upon IL-17A treatment (Figure 3E). Similar results were observed in BGC-823 cell line (Figure S3A–E), indicating that IL-17A activated NF-κB in GC cell lines.

**Figure 4:**
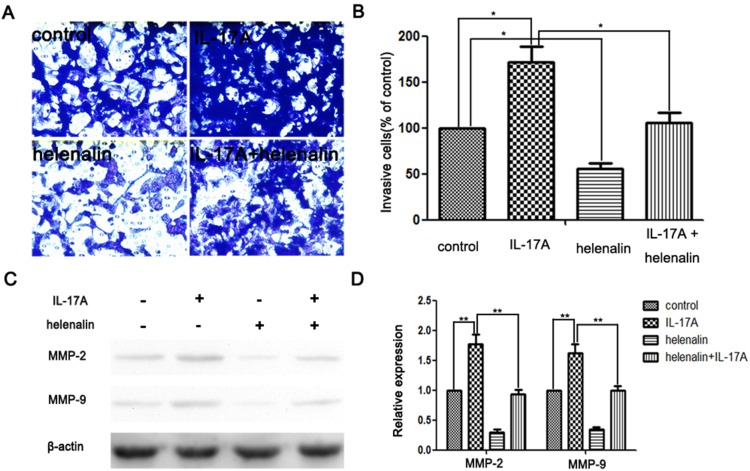
Effects of the NF-κB inhibitor and IL-17A on cell invasion and the expressions of MMP-2 and MMP-9 in AGS cells. (A) 1×10^6^ AGS cells were pretreated with helenalin (5 µM) for 30 min and then incubated in the presence or absence of IL-17A (50 ng/ml) for 24 h. Cellular invasiveness was measured using the transwell invasion assay. (B) The percent invasion rate was expressed as a percentage of control. (C, D) AGS cells were treated and then subjected to western blotting to analyze the protein levels of MMP-2 and MMP-9. Values represent the means ± SD of three independent experiments performed in triplicate. *p<0.05 and **p<0.01 compared with the control group.

Furthermore, when helenalin (5 µM) (Sigma-Aldrich, St. Louis, MO), a NF-κB inhibitor, was added to AGS or BGC-823 cells medium before IL-17A treatment, the expression levels of MMP-2 and MMP-9 were significantly decreased (Figure 4C–D, Figure S4C–D). The result demonstrated that IL-17A induced MMP-2 and MMP-9 expression in GC cell via NF-κB activation. Accordingly, IL-17A induced GC cell invasiveness could also been blocked by helenalin *in vitro* (Figure 4A–B, Figure S4A–B). Hence, these findings suggest that IL-17A activates NF-κB pathway, subsequently regulates the expression of MMPs, and thereby affects migration and invasiveness of GC cells.

## Discussion

GC is one of the most fatal malignant diseases in the world because of its high recurrence rate after curative therapies [3], [22]. GC is closely correlated with chronic inflammatory diseases, in which plenty of inflammatory cytokines infiltrate. IL-17A is one of the important inflammatory cytokines in the development of many inflammatory diseases and is also frequently detected in tumor microenvironment [23]–[26]. Previous study suggested that the expression levels of IL-17 in the tumor could be an independent prognostic indicator in gastric adenocarcinoma patients [27]. Comparison of survival rates between patients with high and low levels of IL-17 found that patients with high levels of IL-17 had a 5-year survival rate of 47% compared with 84% in patients with low levels [28]. However, the molecular mechanisms for such correlation, which may help us better understand the role of IL-17 in the development and progress of GC, remain to be elucidated.

In the present study, we found that IL-17A promoted the invasion of GC cells. Metastasis is one of the leading causes of cancer-related death among GC patients. Degradation of the ECM of blood or lymph vessels is critical to metastasis, because loss of the ECM allows cancer cells to invade the blood or lymphatic system and spread to other tissues and organs [29]. MMPs, especially MMP-2 and MMP-9, are responsible for degrading the ECM [19], [29]. IL-17A has been reported to promote the invasion of cancer cells via upregulating the expression of MMP-2 and MMP-9 [17], [26]. It is generally accepted that MMP activities are inhibited by TIMPs to prevent extensive ECM degradation [29]. To clarify the mechanism of action of IL-17A, we investigated whether the promoting effect of IL-17A on cell invasion is through regulation of the expressions of MMP-2, MMP-9, TIMP-1 and TIMP-2. Our results showed that IL-17A elevated the expressions and activities of MMP-2 and MMP-9, and downregulated the expressions of TIMP-1 and TIMP-2, which further explained that the IL-17A promoted cell migration in the scratch wound healing assay. Therefore, the pro-metastasis effect of IL-17A on GC might be through regulating MMP/TIMP balance.

NF-κB has been reported as a downstream target of IL-17A signaling pathway in many cells [17], [20], [21], which is able to upregulate MMP-2 and MMP-9 expressions [14], [17]. And IL-17A was also reported to increase the expression of MMPs via activating NF-κB pathway in many cells [14], [17]. So we investigated whether the upregulation of MMP-2 and MMP-9 induced by IL-17A is through activating NF-κB pathway. The results showed IL-17A promoted nuclear translocation of the p65 and p50 subunits of GC cells and subsequently upregulated the expression of MMP-2 and MMP-9. This effect could be effectively blocked by NF-κB inhibitor, suggesting that the activation of NF-κB is the potential mechanism to upregulate MMP-2 and MMP-9 by IL-17A.

In conclusion, our findings suggested that IL-17A was able to promote the migration and invasiveness of GC cells by activating NF-κB pathway, which subsequently upregulated the expressions of MMP-2 and MMP-9 and thereby affected migration and invasiveness of GC cells. Further characterization of the effect of IL-17A on GC invasion and metastasis may lead to the identification of new diagnostic markers and therapeutic targets.

## Supporting Information

Figure S1 **The effect of IL-17A on the proliferation of BGC-823, SGC-7901 and AGS cells.**
(TIF)

Figure S2 **IL-17A promotes the expressions of MMP-2 and MMP-9 and suppresses the expressions of TIMP-1 and TIMP-2 in SGC-7901 cells.** (A) Expressions of MMPs in SGC-7901 cells were compared by western blotting between cells treated with and without IL-17A (50 ng/ml) for 24 h. (B) Quantification of the protein levels of MMP-2 and MMP-9. Values represent the means ± SD of three independent experiments performed in triplicate. *p<0.05, compared with the control group.
(TIF)

Figure S3 **IL-17A activates NF-κB in BGC-823 cells.** (A) Western blotting analysis was used to detect overall p50, p65, p52, c-Rel and RelB expression in BGC-823 cells treated with IL-17A (50 ng/mL) at indicated time points. (B) Quantification of the protein levels of overall p50, p65, p52, c-Rel and RelB. (C) Western blotting analysis was used to detect nuclear p50, p65, p52, c-Rel and RelB expression in BGC-823 cells treated with IL-17A (50 ng/mL) at indicated time points. (D) Quantification of the protein levels of nuclear p50, p65, p52, c-Rel and RelB. (E) The relative ratio of nuclear to overall fraction of p50, p65, p52, c-Rel and RelB. Values represent the means ± SD of three independent experiments performed in triplicate. *p<0.05, compared with the control group.
(TIF)

Figure S4 **Effects of helenalin and IL-17A on cell invasion and the expressions of MMP-2 and MMP-9 in BGC-823 cells.** (A) 1×10^6^ BGC-823 cells were pretreated with helenalin (5 µM) and then incubated in the presence or absence of IL-17A (50 ng/mL) for 24 h. Cellular invasiveness was measured using the transwell invasion assay. (B) The percent invasion rate was expressed as a percentage of control. (C, D) The protein levels of MMP-2 and MMP-9 were detected by western blotting, when BGC-823 cells were treated with helenalin and/or IL-17A. Values represent the means ± SD of three independent experiments performed in triplicate. *p<0.05, compared with the control group.
(TIF)
