# Supplementary figures and images for: Interleukin 17A Promotes Gastric Cancer Invasiveness via NF-κB Mediated Matrix Metalloproteinases 2 and 9 Expression
Source: PLoS One. 2014 Jun 6;9(6):e96678. doi: 10.1371/journal.pone.0096678 (PMC4048176; doi:10.1371/journal.pone.0096678)

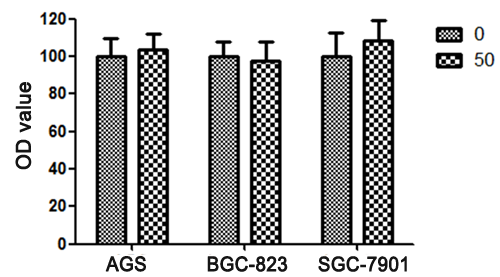

Supplement: Figure S1 — The effect of IL-17A on the proliferation of BGC-823, SGC-7901 and AGS cells. (TIF) [file pone.0096678.s001.tif]

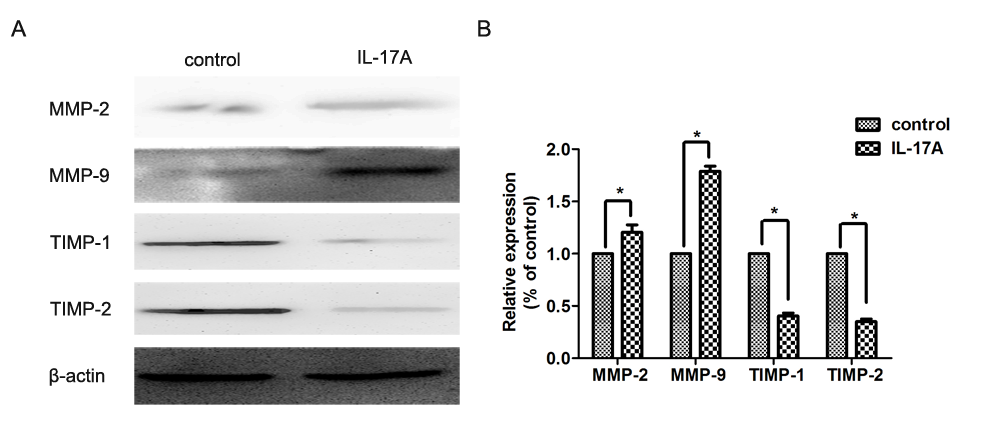

Supplement: Figure S2 — IL-17A promotes the expressions of MMP-2 and MMP-9 and suppresses the expressions of TIMP-1 and TIMP-2 in SGC-7901 cells. (A) Expressions of MMPs in SGC-7901 cells were compared by western blotting between cells treated with and without IL-17A (50 ng/ml) for 24 h. (B) Quantification of the protein levels of MMP-2 and MMP-9. Values represent the means ± SD of three independent experiments performed in triplicate. *p<0.05, compared with the control group. (TIF) [file pone.0096678.s002.tif]

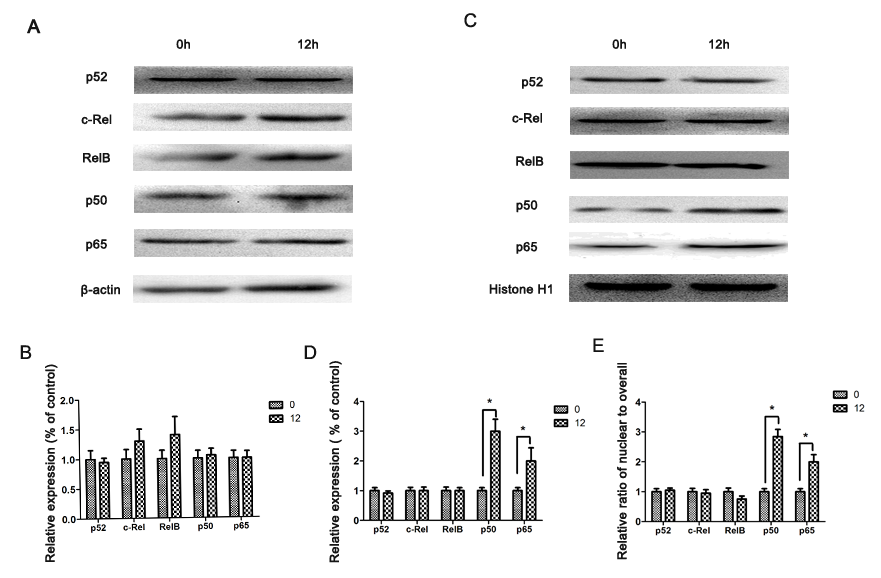

Supplement: Figure S3 — IL-17A activates NF-κB in BGC-823 cells. (A) Western blotting analysis was used to detect overall p50, p65, p52, c-Rel and RelB expression in BGC-823 cells treated with IL-17A (50 ng/mL) at indicated time points. (B) Quantification of the protein levels of overall p50, p65, p52, c-Rel and RelB. (C) Western blotting analysis was used to detect nuclear p50, p65, p52, c-Rel and RelB expression in BGC-823 cells treated with IL-17A (50 ng/mL) at indicated time points. (D) Quantification of the protein levels of nuclear p50, p65, p52, c-Rel and RelB. (E) The relative ratio of nuclear to overall fraction of p50, p65, p52, c-Rel and RelB. Values represent the means ± SD of three independent experiments performed in triplicate. *p<0.05, compared with the control group. (TIF) [file pone.0096678.s003.tif]

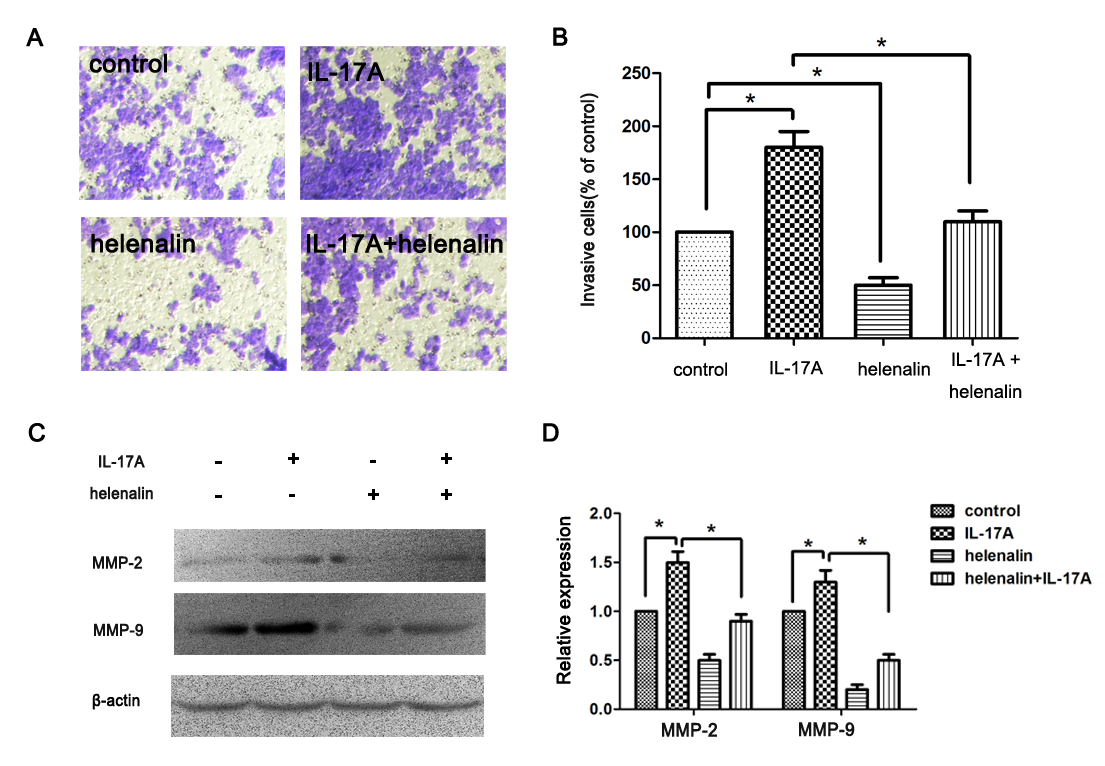

Supplement: Figure S4 — Effects of helenalin and IL-17A on cell invasion and the expressions of MMP-2 and MMP-9 in BGC-823 cells. (A) 1×10^6^ BGC-823 cells were pretreated with helenalin (5 µM) and then incubated in the presence or absence of IL-17A (50 ng/mL) for 24 h. Cellular invasiveness was measured using the transwell invasion assay. (B) The percent invasion rate was expressed as a percentage of control. (C, D) The protein levels of MMP-2 and MMP-9 were detected by western blotting, when BGC-823 cells were treated with helenalin and/or IL-17A. Values represent the means ± SD of three independent experiments performed in triplicate. *p<0.05, compared with the control group. (TIF) [file pone.0096678.s004.tif]
